# The inflammatory protein Pentraxin 3 in cardiovascular disease

**DOI:** 10.1186/s12979-016-0080-1

**Published:** 2016-08-24

**Authors:** Francesco Fornai, Albino Carrizzo, Maurizio Forte, Mariateresa Ambrosio, Antonio Damato, Michela Ferrucci, Francesca Biagioni, Carla Busceti, Annibale A. Puca, Carmine Vecchione

**Affiliations:** 1 Department of Translational Research and New Technologies in Medicine and Surgery, University of Pisa, Pisa, Italy; 2 I.R.C.C.S. Neuromed, Pozzilli, IS Italy; 3 Vascular Physiopathology Unit, I.R.C.C.S. Multimedica, Milan, Italy; 4 Department of Medicine and Surgery, University of Salerno, Via S. Allende, Baronissi, SA 84081 Italy

**Keywords:** Pentraxin 3, Acute phase protein of inflammation, Cardiovascular diseases, Myocardial infarction, Atherosclerosis, Angiogenesis

## Abstract

The acute phase protein Pentraxin 3 (PTX3) plays a non-redundant role as a soluble pattern recognition receptor for selected pathogens and it represents a rapid biomarker for primary local activation of innate immunity and inflammation. Recent evidence indicates that PTX3 exerts an important role in modulating the cardiovascular system in humans and experimental models. In particular, there are conflicting points concerning the effects of PTX3 in cardiovascular diseases (CVD) since several observations indicate a cardiovascular protective effect of PTX3 while others speculate that the increased plasma levels of PTX3 in subjects with CVD correlate with disease severity and with poor prognosis in elderly patients.

In the present review, we discuss the multifaceted effects of PTX3 on the cardiovascular system focusing on its involvement in atherosclerosis, endothelial function, hypertension, myocardial infarction and angiogenesis. This may help to explain how the specific modulation of PTX3 such as the use of different dosing, time, and target organs could help to contain different vascular diseases. These opposite actions of PTX3 will be emphasized concerning the modulation of cardiovascular system where potential therapeutic implications of PTX3 in humans are discussed.

## Background

**Fig. 1:**
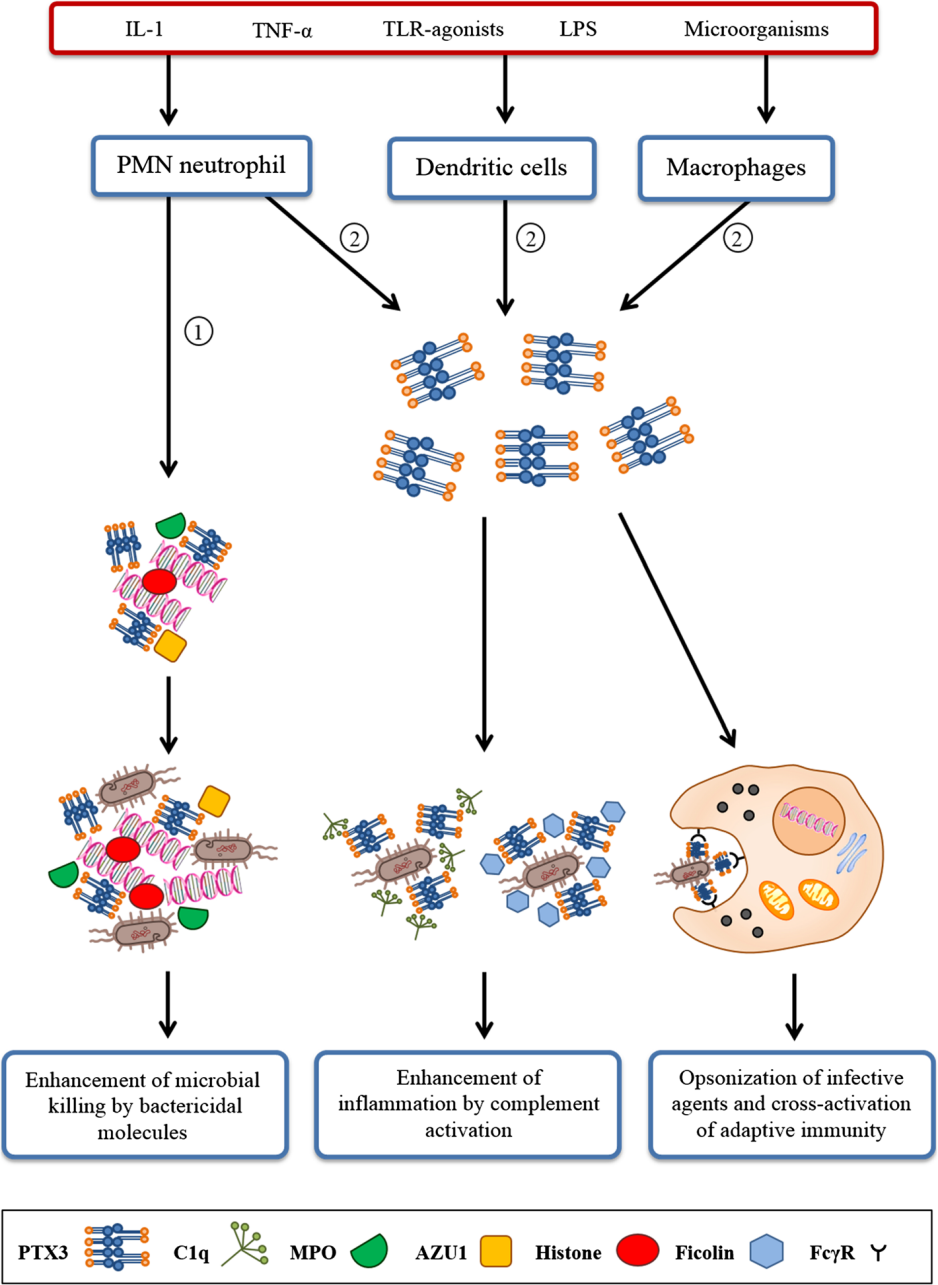
Activity of PTX3 in innate immunity. PTX3 represents the humoral arm of the innate immunity. Inflammatory cytokines, Toll-like receptors (TLRs), microorganisms and microbial moieties stimulate secretion of PTX3 by polymorphonuclear (PMN) neutrophils, macrophages, and dendritic cells. (**1**) Release of PTX3 by PMN neutrophils occurs quickly and casts an immediate defensive response. In fact, these cells possesscytosolic granules containing a stored, ready- to-release, pool of PTX3. (**2**) Macrophages and dendritic cells are other effectors of the innate immunity, which neo-synthesize PTX3 upon stimulation. This newly synthesized pool of PTX3cells is responsible for a slower response to infective agents, which might persist even several days. Released PTX3 regulates inflammatory reactions by acting through several pathways/mechanisms: I) PTX3 released by PMN neutrophils localizesat level of neutrophil extracellular traps (NETs). NETs represent an extracellular fibrillary network, where some nuclear components, such as DNA and histones, are variously assembled with bactericidal proteins, such as azurocidin1(AZU1) andmyeloperoxidase(MPO). Within NETs, PTX3 and anti-microbial molecules converge and cooperate to enhance binding and killing of infective agents. II) PTX3 released in the extracellular space binds to specific microbial ligands and activates the complement cascade through interaction with C1q particles (classical pathway) or ficolins and mannose-binding lectins (lectin-mediated pathway). PTX3-induced complement activation enhances the inflammatory response. III) Finally, extracellular PTX3 opsonizes microorganisms binding to specific molecules on the cell surface (i.e. zymosan on *Aspergillusfumigatus*) and, in turn, it is recognized by Fcgamma receptors expressed by phagocytic cells, thus promoting microbial clearance. The interaction of PTX3with Fcgamma receptors indicates the antibody-like function of PTX3 and underlies its functional overlapping between innate and adaptive immunity during inflammation. (*FcγR* Fcgamma receptor, *IL-1* interleukin-1, *LPS* lipopolysaccaride, *PTX3* pentraxin 3, *TNF-alpha* tumor necrosis factor-alpha)

PTX3 belongs to a superfamily of phylogenically conserved multimeric proteins, which includes short and long pentraxins [1, 2]. All these proteins play a critical role in innate immunity and they are generally considered acute phase immunity proteins [2, 3]. However, their effects which are grounded on the modulation of the cardiovascular system influence a variety of phenomena such as inflammation, angiogenesis, tumorigenesis, cell adhesion [4, 5]. Short and long pentraxins possess a different protein size and they are synthesized by different genes under the influence of different gene promoters. In fact, short and long pentraxins are produced by different cell types in response to different stimuli and possess different molecular targets (Fig. 1). Among short pentraxins C-reactive protein (CRP) is well known. This protein is produced by hepatocytes and other cell types during inflammation. Release of CRP is induced by pro-inflammatory cytokines (mainly interleukin-6, IL-6). Similarly, a short pentraxin is the serum amyloid P-component (SAP), which is solely synthesized by hepatocytes [6].

In contrast, PTX3 belongs to long pentraxins and, as mentioned above, it possesses multifaceted properties extending beyond the fields of immunity and inflammation to CVD [7, 8].

Interestingly, its multiple roles can be considered as site-specific since its production occurs in a variety of cell types, including endothelial cells, fibroblasts, hepatocytes, and monocytes. Moreover, during an acute phase response induced by LPS, PTX3 is expressed in a variety of organs, most prominently in the heart and skeletal muscle, unlike SAP, which is produced only in the liver [9]. Thus, this specific production pattern of PTX3, explains its involvement in multiple cardiovascular disorders.

The present review focuses on findings in humans and it discusses the molecular mechanisms encompassing experimental models and human beings.

## PTX3 in cardiovascular diseases

Cardiovascular diseases (CVD) represent the major cause of death in the developed world [10]. In addition to well established risk factors such as diabetes, hypertension, dyslipidemia, recent evidence indicates that as it occurs for molecules belonging to the acute phase of inflammation, PTX3 may play a key role in the onset and progression of CVD [11–13].

Thus, PTX3 represents a specific and sensitive marker connecting inflammation with CVD since it is expressed and released by most cell compartments involved in the onset and progression of CVD.

PTX3 is involved in a variety of molecular mechanisms leading to vascular damage and its elevated plasma levels represent a significant predictor of frailty in elderly hypertensive patients [14]. In fact, blood vessels produce large amounts of PTX3 during inflammation, and the level of circulating PTX3 increases in several pathological conditions affecting the cardiovascular system [15, 16].

Here, we highlight the bad or good associations between PTX3 and CVD in humans. In addition, in experimental models we reported the mechanisms by which this inflammatory protein may exert itson the cardiovascular actions.

### Atherosclerosis

Although cholesterol accumulation intimal layer is the major pathological feature of atherogenesis, growing evidence suggests that the inflammatory state represents the major detrimental factor for the progression of atherosclerosis both in young and in elderly subjects. In fact, the earliest stage of atherosclerotic damage is characterized by infiltration of macrophages and T-lymphocytes, which are progressively activated during the course of the damage [17, 18]. Based on the kind of cell phenotype being recruited (macrophages and vascular cells), which is known to produce PTX3, this protein was investigated as a potential modulator of atherosclerosis.

In fact, human sclerotic mammary arteries possess high levels of PTX3, which is mainly localized within endothelial cells and macrophages [19, 20]. In addition, smooth muscle cells, treated with inflammatory stimuli such as oxidized lipoproteins, increase their PTX3 mRNA level. This effect leads to a vascular acute-phase-response activating the classic pathway of complement [21], which represents one of the most important mechanisms leading to chemo tactic and pro-inflammatory effects. The link between PTX3 and complement system was confirmed by the occurrence of high PTX3 level within atherosclerosis-related coronary arterial thrombi that are mainly constituted by resident macrophages, neutrophils and foam cells [22].

Recently, epidemiological and clinical data candidate PTX3 as a valid biomarker for atherosclerosis [19, 22]. In particular, PTX3 plays a role in the regulation of innate resistance to inflammatory reactions, it is strongly expressed in atherosclerotic arteries [23] and its high plasma levels were found to be related with severity of coronary atherosclerosis [24]. Accordingly, PTX3 levels are significantly increased in patients with carotid stenosis [25], as well as in patients with acute coronary syndrome [26], in whom PTX3 is a candidate biomarker for plaque vulnerability [27]. Finally, PTX3 has been indicated also as a marker of neo-intimal thickening after vascular injury, since elevated levels of PTX3 have been found in patients with atherosclerosis, after 15 min from coronary stenting [28].

Although the presence of PTX3 in human atherosclerotic damage is well defined, its causal role in the onset and progression of atherosclerosis, remains unclear. Here, data obtained in experimental models are reviewed aimed at suggesting the mechanistic role of PTX3 in atherogenesis.

In particular, it has been reported that PTX3, by interacting with P-selectin, a cell-adhesion molecule involved in the tethering and rolling of leukocytes and platelets on activated endothelial cells, attenuates leukocytes recruitment at the site of inflammation [29]. These data clearly indicate a protective role of PTX3 in atherosclerosis. In keeping with this, in a double knockout mouse model for PTX3 and apolipoprotein E (ApoE), macrophages infiltration was enhanced, and a dramatic increase of atherosclerosis was reported. The molecular analysis indicates a dramatic rise in the pattern of inflammatory genes within vascular wall concomitant with up-regulation of pro-inflammatory mediators such as chemokines, cytokines, adhesion molecules, E-selectin and transcription factors, such as NF-kB [30].

In contrast, PTX3 was also reported to induce deleterious effects in the pathogenesis of atherothrombosis [31]. On this regard, it has been demonstrated that PTX3 increases the tissue factor (TF) expression in mononuclear and endothelial cells. The increased level of TF, the main orchestrator of the coagulation cascade, causes the thrombus formation, a feature of atherosclerosis [32]. PTX3 might also interfere with plaque stability by binding the fibroblast growth factor 2 (FGF2), that play a role in proliferation and migration of smooth muscle cells [33]. This evidence suggests a bad cop for PTX3, which promotes lesion progression through a stronger innate immune response. Recently, PTX3 has been proposed as a potential therapeutic target to limit the development of atherosclerosis. In fact, by using a cell model of atherosclerosis, it was shown that suppression of PTX3 reduces inflammation and apoptosis mediated by the IkB kinase (IKK)/IkB/nuclear factor-kB (NF-kB) pathway [34].

Actually, the role of PTX3 in the atherosclerosis is not well-understood. It will be interesting to examine the role of the protein in complement activation during atherogenesis to define the specific role of PTX3 in the atherosclerosis. Overall, PTX3 seems to exert detrimental effects on atherosclerotic process since it promotes plagues formation and leads to an amplification of vascular inflammation such as to be candidate it as a new possible biomarker for plaque vulnerability. However, only in those conditions in which it is able to reduce macrophages infiltration and the expression of adhesion molecules it exerts beneficial effects.

### Endothelial dysfunction and hypertension

Endothelial dysfunction is a typical trait of several cardiovascular disorders including arterial hypertension. The impairment of the nitric oxide pathway and the enhanced smooth muscle vasoconstriction represent the main mechanisms leading to endothelial dysfunction in hypertension [35, 36].

A growing body of evidence attributes to local and systemic inflammation a key role in the development of endothelial dysfunction, thus suggesting a key role for acute phase proteins [37, 38].

On this regard, high plasma levels of the inflammatory molecule PTX3 were associated with endothelial dysfunction in different human diseases. Patients with chronic kidney diseases and with preeclampsia, a multisystemic disorder associated with hypertension [39–41], show elevated PTX3 plasma levels which were correlated with the severity of endothelial dysfunction [39, 40, 42].

In addition, elevated plasma level of PTX3 have been found in patients with high systolic and diastolic blood pressure levels [16, 43] and in elderly hypertensive patients with high 24-h blood pressure levels [14]. In pulmonary arterial hypertension, PTX3 has been proposed as a more specific and sensitive biomarker compared with brain natriuretic peptide, which so far, was considered the gold standard marker for pulmonary hypertension [44, 45].

The evidence described so far in humans suggests a potential role of PTX3 in regulating vascular homeostasis. In experimental models, the absence of PTX3 is accompanied by massive suppression of tissue inflammation, cell injury and it determines decreased lethality after reperfusion of an ischemic superior mesenteric artery in mice, thus demonstrating that PTX3 is relevant in conditioning tissue damage [46]. In a recent study by Carrizzo et al. we investigated the molecular mechanisms recruited by PTX3 to induce damage of mesenteric artery. In particular, exogenous administration of PTX3 in mice impaired endothelial function in resistance vessels through a P-selectin/matrix metalloprotease*1* pathway, thus producing morphological alterations of endothelial cells and disruption of nitric oxide signalling. These effects were associated with an increase in blood pressure in vivo. It is worth to be mentioned that PTX3, and its mediators of vascular damage are present at higher levels within plasma of hypertensive patients compared with normotensive subjects [47]. Based on these data, PTX3 could be considered as a novel biomarker for hypertension and a new target for future therapeutic strategy aimed to contain endothelial dysfunction and associated CVD.

### Myocardial infarction

Myocardial infarction (MI) continues to be a significant cause of mortality and morbidity worldwide [48]. Over the past 50 years, it has become clear that the cascade of thrombotic events following atherosclerotic plaque rupture causes occlusion of the coronary artery, interrupting blood supply and oxygen supply to myocardium, thus producing infarction. The significance of occurrence of PTX3 expression in the heart from normal and hypertrophy human cardiac cells still needs to be clarified for its physiological pathological role [49, 50].

In older adults it was reported an association between PTX3 plasma levels, CVD and all causes of death, independently from CVD risk factors [25]. In particular, plasma level of PTX3 increases in patients with acute myocardial infarction (AMI) after about 7 h from the onset of symptoms, with a decrease at baseline levels after three days [50, 51]. Based on these data, PTX3 seems to be at the same time an early indicator of AMI, and also a prognostic marker for the outcomes of heart diseases. PTX3 level also predicts cardiac events in patients with heart failure (HF), suggesting a stratification of HF patients based on PTX3 plasma level [52]. This is fully supported by the identification of coronary circulation as the main source of PTX3 in HF patients with normal ejection fraction [53]. Moreover, in patients with MI, ST tract elevation and with chronic HF, PTX3, but not other cardiac biomarkers, modulating complement components, predicted 3 months mortality, after adjustment for major risk factors [12, 28, 54].

Recently, PTX3 was suggested to be a prognostic marker also in patients with coronary artery diseases after drug stent implantation, and in patients with angina pectoris [55–57], in whom adverse cardiac events were related with PTX3 plasma levels [58].

These studies in humans, do not allow to establish the specific action of PTX3 in the myocardium. Thus, to better characterize the cardiac action of PTX3, here we report data obtained in transgenic *ptx3* deficient mice. After cardiac ischemia- reperfusion injury, *ptx3* deficient mice develop increased myocardial damage, characterized by no-reflow area, increased neutrophil infiltration apoptotic cells and decreased number of capillaries. In addition, in the myocardium of these mice the C3 complement component increased focally being related with the area of damaged myocardium. The evidence that in PTX3-KO mice the administration of exogenous PTX3 reduces complement C3 deposition rescuing the phenotype, highlights the cardio-protective effect of PTX3, through the modulation of the complement cascade [59].

The discovery of PTX3 in the myocardial tissue and the characterization of its role lead to propose PTX3 as an early indicator of myocyte irreversible injury in ischemic cardiomyopathy. In addition, considering the local production and the rapid change in plasma concentration, PTX3 could be considered a novel potential biomarker of myocardial infarction.

### Angiogenesis

Additional activities of PTX3 other than those related to innate immunity and inflammation have been described, such as extracellular matrix deposition (ECM), tissue remodeling, and angiogenesis. Several studies demonstrate that PTX3 is required for the secretion of extracellular matrix. This is critical for a number of functions such as maturation of the oophorus follicle, which explains why in PTX3-deficient mice sterility is observed. This condition seems to be related to a defective PTX3-dependent incorporation of the glycosaminoglycan hyaluronic acid into the matrix of the cumulus oophorus [60]. A similar molecular composition of the ECM, containing hyaluronan (HA), tumor necrosis factor-stimulated gene-6 (TSG-6), and inter-α-inhibitor (IαI), is observed in rheumatoid arthritis and other inflammatory infiltrates, suggesting a role of PTX3 also in these conditions [61].

Interestingly, PTX3 regulates angiogenesis via FGF2, thus interfering with a variety of conditions, including ontogenesis, growth, inflammation, tissue repair, atherosclerosis, and tumors [62]. The PTX3/FGF2 interaction prevents angiogenesis. Beyond the anti-angiogenetic activity, the inhibitory effects of PTX3 on FGF2 possess a therapeutic rationale in the treatment of re-stenosis, the progressive occlusion of the coronary artery which occurs often following angioplastic surgery, due to anomalous FGF2-dependent proliferation and accumulation of smooth muscle cells on the vessel wall [63]. On the other hand, angiogenesis involves remodeling of ECM. In this respect, FGF2 modulates degradation of the ECM by inducing expression of urokinase-type plasminogen activator (uPA) and plasminogen activator inhibitor (PAI)-1 on endothelial cell surface, with opposite effects on matrix metalloprotease (MMP) activity. Moreover, FGF2 regulates expression and distribution of several integrins and cadherins on the plasma membrane of endothelial cells, thus promoting cell scattering or adhesion in different stages of angiogenesis [64]. Finally, in PTX3 deficient mice models a dramatic reduction of vascular endothelial growth factor receptor 2 (VEGFR2)occurs. This contributes to worsening the outcome of cerebral ischemia associated with a reduction of vessels formation [65].

### Retinal vasculature

Recently, some studies have investigated on the role of the PTX3 on the modulation of retinal vasculature [66]. In particular, Moon Woo and colleagues have demonstrated that human retinal pigment epithelial cells are the major source of PTX3 following pro-inflammatory cytokines stimulation and that its release exerts an important role in retinal injury against inflammation and infection. Diabetic Retinopathy (DR) represents the well-known microvascular complication of diabetes [67]. A recent study has demonstrated that PTX3 levels were significantly elevated in diabetic patients with DR compared to diabetic patients without DR and normal subjects. This association is correlated with an important retinal microvascular dysfunction, characterized by capillary leakage or closure, leading to ischemia [67, 68]. This study suggests that plasma PTX3 may be a better predictor for DR than CRP in diabetic patients. Moreover, Noma et al. have highlighted the relationship between PTX3 and retinal diseases such as age-related macular degeneration (AMD) and retinal vascular occlusion [69]. On this regards, it has been reported an interaction between PTX3 and the complement regulator factor H (CFH), a soluble molecule of the alternative pathway of the complement system involved in AMD pathogenesis. The authors hypothesize that aberrant expression of PTX3 could be associated with pathophysiology of AMD [69] because there is a loss of control of CFH activity. PTX3 has been evaluated also in the retinal vein occlusion (RVO), the second most common retinal vascular disease after diabetic retinopathy. This study demonstrate that PTX3 could be used as a useful diagnostic biomarker since patients with RVO have high plasma level of PTX3 [70]. Finally, during 2016 Zhou and Hu suggest that the use of PTX3 could be used as an anti-angiogenic molecule because PTX3 interacts specifically with FGF2 factor reducing the proliferative diabetic retinopathy thus ameliorating DR condition [68]. Although there are not specific evaluation of molecular signaling recruited by PTX3 in retinal vasculature, it could be represent another important field in which is possible to act modulating PTX3 to obtain favorable vascular results.

### Genetics and epigenetics

Epigenetic modulation of PTX3 gene in cardiovascular diseases is increasingly emerging. A candidate modulator of PTX3 is TNFα. In fact, TNFα controls the expression of PTX3 by human adipose tissue, being potentially implicated in the development of atherosclerosis [71], and administration of antibodies against TNFα lowers the levels of PTX3 transcripts in Kawasaki disease (KD) patients with intravenous immunoglobulin resistance, thus reducing the risk to develop coronary artery aneurysms [72].

Another very recent study demonstrated that PTX3 gene is over expressed in the presence of Nogo-B, a member of the reticulon 4 protein family, which is critical in vessel regeneration [73, 74]. In rheumatoid synoviocytes transcription of the PTX3 messenger RNA (mRNA) was found to be directly promoted by serum amyloid A [75]. Moreover, plasma analysis and gene expression profile revealed that PTX3 might be involved in the remote cardioprotective effects observed in mice after subcutaneous transplantation of heme oxygenase-1-overexpressing mouse mesenchymal stem cells [76]. Interestingly, in experimental brain stroke PTX3 is found locally increased in response to the pro-inflammatory cytokine interleukin-1 and it contributes to brain recovery by promoting glial scar formation and resolution of edema [77]. It is worth to be mentioned that the epigenetic regulation of long pentraxin 3 was demonstrated to recruit the PI3K/Akt pathway [78]. Remarkably, it was shown that HDL may increase the expression of PTX3 via activation of the PI3K/Akt pathway [79], which discloses a further beneficial effects of PTX3 under the regulation of HDL.

## Conclusion

**Fig. 2:**
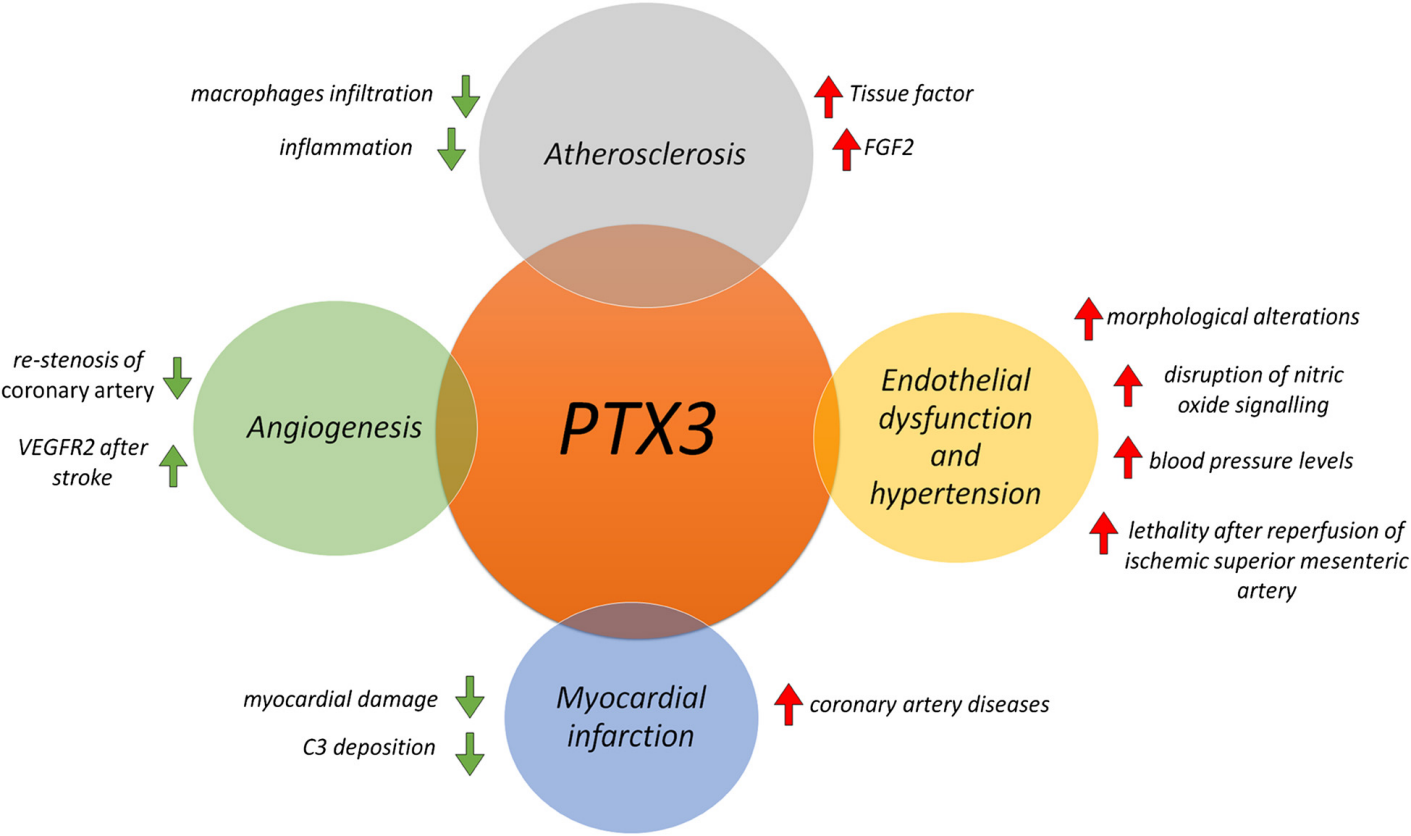
Schematic representation of the detrimental or beneficial effect of PTX3 in cardiovascular diseases. *Red arrow* indicates a deleterious effect evoked by PTX3, in contrast to the *Green arrow*, which indicates the beneficial effect of the protein. The mechanisms through which the PTX3 exerts its cardiovascular effects are described on the side of the arrows

The multiple effects played by PTX3 are now of growing interest. Here we try to explain the mechanistic interactions of PTX3 in cardiovascular diseases discussing recent evidence coming from both cardiovascular and systemic studies showing the fine tuning of time, space, and organ context, which determine the final outcome of PTX3 effects including either detrimental or beneficial effects (Fig. 2). Several studies have suggested PTX3 as a therapeutic tool for cardiovascular disorders although contradictory findings leave this point unresolved.

Nevertheless, considering that the ptx3-deficient mice have greater myocardial lesions following the coronary artery ligation/reperfusion damage due to activation of C1q [59], the PTX3 stimulation could be used as therapeutic tool to reduced AMI damage. Moreover, since HDL induces PTX3 through PI3K/Akt axis [80] exerting an atheroprotective effect, the positive modulation of PTX3 could counterbalancing the over activation of a proinflammatory atherogenic cascade thus protecting the vascular wall. Again, based on the interaction between PTX3 and complement factor H that contribute to maintain the retinal immunohomeostasis [81], the stimulation of PTX3 could reduce the pathophysiology of macular degeneration. Finally, in keeping with the detrimental vascular effects of PTX3 on endothelium, mediated by the activation of P-Selectin/MMP1, we wish to emphasize that PTX3 may represent a novel target to be blocked to protect blood vessels. Based on these data, we feel that the balance between the good and the bad cops of PTX3 must be kept in mind both kind of effects when planning further developments in human patients. In particular, the vascular-based therapeutic approach should be limited to block the vascular effects of PTX3 without affecting its beneficial effects in other organs. Alternatively one might theoretically plan to combine opposite actions in different systems. Opposite manipulations of PTX3 for future therapeutic strategies should be planned to block selectively PTX3-induced molecular cascade in blood vessel, while concomitantly increasing its effects in other selective district. These issues represent a challenge for future PTX3-based drug development.

## Abbreviations

AMI: Acute myocardial infarction
ApoE: Apolipoprotein E
CRP: C-reactive protein
CVD: Cardiovascular diseases
ECM: Extracellular matrix deposition
FGF2: Fibroblast growth factor 2
HF: Heart failure
IKK: IkB kinase
IL-6: Interleukin-6
LPS: Lipopolysaccharides
MI: Myocardial infarction
MMP: Matrix metalloprotease
NF-kB: Nuclear factor kappa-light-chain-enhancer of activated B cells
PTX3: Pentraxin 3
SAP: Serum amyloid protein
VEGFR2: Vascular endothelial growth factor receptor 2

## References

[1] Garlanda C, Bottazzi B, Bastone A, Mantovani A (2005). Pentraxins at the crossroads between innate immunity, inflammation, matrix deposition, and female fertility. Annu Rev Immunol.

[2] Gewurz H, Zhang XH, Lint TF (1995). Structure and function of the pentraxins. Curr Opin Immunol.

[3] Mantovani A, Garlanda C, Doni A, Bottazzi B (2008). Pentraxins in innate immunity: from C-reactive protein to the long pentraxin PTX3. J Clin Immunol.

[4] Bonacina F, Barbieri SS, Cutuli L, Amadio P, Doni A, Sironi M (2016). Vascular pentraxin 3 controls arterial thrombosis by targeting collagen and fibrinogen induced platelets aggregation. Biochim Biophys Acta.

[5] Bottazzi B, Inforzato A, Messa M, Barbagallo M, Magrini E, Garlanda C (2016). The pentraxins PTX3 and SAP in innate immunity, regulation of inflammation and tissue remodelling. J Hepatol.

[6] Agrawal A, Singh PP, Bottazzi B, Garlanda C, Mantovani A (2009). Pattern recognition by pentraxins. Adv Exp Med Biol.

[7] Fornai F, Carrizzo A, Ferrucci M, Damato A, Biagioni F, Gaglione A (2015). Brain diseases and tumorigenesis: the good and bad cops of pentraxin3. Int J Biochem Cell Biol.

[8] Dubin R, Li YM, Ix JH, Shlipak M, Whooley M, Peralta CA. Associations of Pentraxin-3 with cardiovascular events, incident heart failure and mortality among persons with coronary heart disease: data from the heart and soul study. Circulation. 2011;124. 10.1016/j.ahj.2011.11.007 PMC3273726 22305847

[9] Introna M, Alles VV, Castellano M, Picardi G, DeGioia L, Bottazzi B (1996). Cloning of mouse ptx3, a new member of the pentraxin gene family expressed at extrahepatic sites. Blood.

[10] Fuster V, Mearns BM (2009). The CVD paradox: mortality vs prevalence. Nat Rev Cardiol.

[11] Willerson JT, Ridker PM (2004). Inflammation as a cardiovascular risk factor. Circulation.

[12] Latini R, Maggioni AP, Peri G, Gonzini L, Lucci D, Mocarelli P (2004). Prognostic significance of the long pentraxin PTX3 in acute myocardial infarction. Circulation.

[13] Inoue K, Kodama T, Daida H (2012). Pentraxin 3: a novel biomarker for inflammatory cardiovascular disease. Int J Vasc Med.

[14] Yano Y, Matsuda S, Hatakeyama K, Sato Y, Imamura T, Shimada K (2010). Plasma Pentraxin 3, but not high-sensitivity C-reactive protein, is a useful inflammatory biomarker for predicting cognitive impairment in elderly hypertensive patients. J Gerontol A Biol Sci Med Sci.

[15] Norata GD, Garlanda C, Catapano AL (2010). The long pentraxin PTX3: a modulator of the immunoinflammatory response in atherosclerosis and cardiovascular diseases. Trends Cardiovasc Med.

[16] Parlak A, Aydogan U, Iyisoy A, Dikililer MA, Kut A, Cakir E (2012). Elevated pentraxin-3 levels are related to blood pressure levels in hypertensive patients: an observational study. Anadolu Kardiyol Derg.

[17] Libby P (2002). Inflammation in atherosclerosis. Nature.

[18] Libby P, Okamoto Y, Rocha VZ, Folco E (2010). Inflammation in atherosclerosis: transition from theory to practice. Circ J.

[19] Rolph MS, Zimmer S, Bottazzi B, Garlanda C, Mantovani A, Hansson GK (2002). Production of the long pentraxin PTX3 in advanced atherosclerotic plaques. Arterioscler Thromb Vasc Biol.

[20] Zacho J, Tybjaerg-Hansen A, Nordestgaard BG (2010). C-reactive protein and all-cause mortality--the Copenhagen City Heart Study. Eur Heart J.

[21] Klouche M, Peri G, Knabbe C, Eckstein HH, Schmid FX, Schmitz G (2004). Modified atherogenic lipoproteins induce expression of pentraxin-3 by human vascular smooth muscle cells. Atherosclerosis.

[22] Savchenko A, Imamura M, Ohashi R, Jiang S, Kawasaki T, Hasegawa G (2008). Expression of pentraxin 3 (PTX3) in human atherosclerotic lesions. J Pathol.

[23] Mantovani A, Garlanda C, Bottazzi B (2003). Pentraxin 3, a non-redundant soluble pattern recognition receptor involved in innate immunity. Vaccine.

[24] Nerkiz P, Doganer YC, Aydogan U, Akbulut H, Parlak A, Aydogdu A (2015). Serum pentraxin-3 level in patients who underwent coronary angiography and relationship with coronary atherosclerosis. Med Princ Pract.

[25] Jenny NS, Arnold AM, Kuller LH, Tracy RP, Psaty BM (2009). Associations of pentraxin 3 with cardiovascular disease and all-cause death: the Cardiovascular Health Study. Arterioscler Thromb Vasc Biol.

[26] Eggers KM, Armstrong PW, Califf RM, Johnston N, Simoons ML, Venge P (2013). Clinical and prognostic implications of circulating pentraxin 3 levels in non ST-elevation acute coronary syndrome. Clin Biochem.

[27] Soeki T, Niki T, Kusunose K, Bando S, Hirata Y, Tomita N (2011). Elevated concentrations of pentraxin 3 are associated with coronary plaque vulnerability. J Cardiol.

[28] Kotooka N, Inoue T, Aoki S, Anan M, Komoda H, Node K (2008). Prognostic value of pentraxin 3 in patients with chronic heart failure. Int J Cardiol.

[29] Deban L, Russo RC, Sironi M, Moalli F, Scanziani M, Zambelli V (2010). Regulation of leukocyte recruitment by the long pentraxin PTX3. Nat Immunol.

[30] Norata GD, Marchesi P, Pulakazhi Venu VK, Pasqualini F, Anselmo A, Moalli F (2009). Deficiency of the long pentraxin PTX3 promotes vascular inflammation and atherosclerosis. Circulation.

[31] Shindo A, Tanemura H, Yata K, Hamada K, Shibata M, Umeda Y (2014). Inflammatory biomarkers in atherosclerosis: pentraxin 3 can become a novel marker of plaque vulnerability. PLoS One.

[32] Napoleone E, di Santo A, Peri G, Mantovani A, de Gaetano G, Donati MB (2004). The long pentraxin PTX3 up-regulates tissue factor in activated monocytes: another link between inflammation and clotting activation. J Leukoc Biol.

[33] Bassi N, Zampieri S, Ghirardello A, Tonon M, Zen M, Cozzi F (2009). Pentraxins, anti-pentraxin antibodies, and atherosclerosis. Clin Rev Allergy Immunol.

[34] Qiu L, Xu R, Wang S, Li S, Sheng H, Wu J (2015). Honokiol ameliorates endothelial dysfunction through suppression of PTX3 expression, a key mediator of IKK/IkappaB/NF-kappaB, in atherosclerotic cell model. Exp Mol Med.

[35] Forstermann U, Munzel T (2006). Endothelial nitric oxide synthase in vascular disease: from marvel to menace. Circulation.

[36] Puca AA, Carrizzo A, Ferrario A, Villa F, Vecchione C (2012). Endothelial nitric oxide synthase, vascular integrity and human exceptional longevity. Immun Ageing.

[37] Carnevale D, Pallante F, Fardella V, Fardella S, Iacobucci R, Federici M (2014). The angiogenic factor PlGF mediates a neuroimmune interaction in the spleen to allow the onset of hypertension. Immunity.

[38] Clapp BR, Hingorani AD, Kharbanda RK, Mohamed-Ali V, Stephens JW, Vallance P (2004). Inflammation-induced endothelial dysfunction involves reduced nitric oxide bioavailability and increased oxidant stress. Cardiovasc Res.

[39] Witasp A, Ryden M, Carrero JJ, Qureshi AR, Nordfors L, Naslund E (2013). Elevated circulating levels and tissue expression of pentraxin 3 in uremia: a reflection of endothelial dysfunction. PLoS One.

[40] Hamad RR, Eriksson MJ, Berg E, Larsson A, Bremme K (2012). Impaired endothelial function and elevated levels of pentraxin 3 in early-onset preeclampsia. Acta Obstet Gynecol Scand.

[41] Cozzi V, Garlanda C, Nebuloni M, Maina V, Martinelli A, Calabrese S (2012). PTX3 as a potential endothelial dysfunction biomarker for severity of preeclampsia and IUGR. Placenta.

[42] Suliman ME, Qureshi AR, Carrero JJ, Barany P, Yilmaz MI, Snaedal-Jonsdottir S (2008). The long pentraxin PTX-3 in prevalent hemodialysis patients: associations with comorbidities and mortality. QJM.

[43] Jylhava J, Haarala A, Kahonen M, Lehtimaki T, Jula A, Moilanen L (2011). Pentraxin 3 (PTX3) is associated with cardiovascular risk factors: the Health 2000 Survey. Clin Exp Immunol.

[44] Tamura Y, Ono T, Kuwana M, Inoue K, Takei M, Yamamoto T (2012). Human pentraxin 3 (PTX3) as a novel biomarker for the diagnosis of pulmonary arterial hypertension. PLoS One.

[45] Naito A, Tanabe N, Jujo T, Shigeta A, Sugiura T, Sakao S (2014). Pentraxin3 in chronic thromboembolic pulmonary hypertension: a new biomarker for screening from remitted pulmonary thromboembolism. PLoS One.

[46] Souza DG, Amaral FA, Fagundes CT, Coelho FM, Arantes RM, Sousa LP (2009). The long pentraxin PTX3 is crucial for tissue inflammation after intestinal ischemia and reperfusion in mice. Am J Pathol.

[47] Carrizzo A, Lenzi P, Procaccini C, Damato A, Biagioni F, Ambrosio M (2015). Pentraxin 3 induces vascular endothelial dysfunction through a P-selectin/Matrix metalloproteinase-1 pathway. Circulation.

[48] Okrainec K, Banerjee DK, Eisenberg MJ (2004). Coronary artery disease in the developing world. Am Heart J.

[49] Haibo Liu, Xiaofang Guo, Kang Yao, Chunming Wang, Guozhong Chen, Wei Gao, Jie Yuan, Wangjun Yu, Junbo Ge. Pentraxin-3 Predicts Long-Term Cardiac Events in Patients with Chronic Heart Failure. BioMedResearch International. 2015;1-7. 10.1155/2015/817615 PMC4633533 26579541

[50] Peri G, Introna M, Corradi D, Iacuitti G, Signorini S, Avanzini F (2000). PTX3, a prototypical long pentraxin, is an early indicator of acute myocardial infarction in humans. Circulation.

[51] Duran S, Duran I, Kaptanagasi FA, Nartop F, Ciftci H, Korkmaz GG (2013). The role of pentraxin 3 as diagnostic value in classification of patients with heart failure. Clin Biochem.

[52] Suzuki S, Takeishi Y, Niizeki T, Koyama Y, Kitahara T, Sasaki T (2008). Pentraxin 3, a new marker for vascular inflammation, predicts adverse clinical outcomes in patients with heart failure. Am Heart J.

[53] Matsubara J, Sugiyama S, Nozaki T, Sugamura K, Konishi M, Ohba K (2011). Pentraxin 3 is a new inflammatory marker correlated with left ventricular diastolic dysfunction and heart failure with normal ejection fraction. J Am Coll Cardiol.

[54] Latini R, Gullestad L, Masson S, Nymo SH, Ueland T, Cuccovillo I (2012). Pentraxin-3 in chronic heart failure: the CORONA and GISSI-HF trials. Eur J Heart Fail.

[55] Inoue K, Sugiyama A, Reid PC, Ito Y, Miyauchi K, Mukai S (2007). Establishment of a high sensitivity plasma assay for human pentraxin3 as a marker for unstable angina pectoris. Arterioscler Thromb Vasc Biol.

[56] Helseth R, Solheim S, Opstad T, Hoffmann P, Arnesen H, Seljeflot I (2014). The time profile of Pentraxin 3 in patients with acute ST-elevation myocardial infarction and stable angina pectoris undergoing percutaneous coronary intervention. Mediators Inflamm.

[57] Matsui S, Ishii J, Kitagawa F, Kuno A, Hattori K, Ishikawa M (2010). Pentraxin 3 in unstable angina and non-ST-segment elevation myocardial infarction. Atherosclerosis.

[58] Haibo L, Xiaofang G, Chunming W, Jie Y, Guozhong C, Limei Z (2014). Prognostic value of plasma pentraxin-3 levels in patients with stable coronary artery disease after drug-eluting stent implantation. Mediators Inflamm.

[59] Salio M, Chimenti S, De Angelis N, Molla F, Maina V, Nebuloni M (2008). Cardioprotective function of the long pentraxin PTX3 in acute myocardial infarction. Circulation.

[60] Salustri A, Garlanda C, Hirsch E, De Acetis M, Maccagno A, Bottazzi B (2004). PTX3 plays a key role in the organization of the cumulus oophorus extracellular matrix and in in vivo fertilization. Development.

[61] Day AJ, de la Motte CA (2005). Hyaluronan cross-linking: a protective mechanism in inflammation?. Trends Immunol.

[62] Presta M, Oreste P, Zoppetti G, Belleri M, Tanghetti E, Leali D (2005). Antiangiogenic activity of semisynthetic biotechnological heparins: low-molecular-weight-sulfated Escherichia coli K5 polysaccharide derivatives as fibroblast growth factor antagonists. Arterioscler Thromb Vasc Biol.

[63] Camozzi M, Zacchigna S, Rusnati M, Coltrini D, Ramirez-Correa G, Bottazzi B (2005). Pentraxin 3 inhibits fibroblast growth factor 2-dependent activation of smooth muscle cells in vitro and neointima formation in vivo. Arterioscler Thromb Vasc Biol.

[64] Leali D, Inforzato A, Ronca R, Bianchi R, Belleri M, Coltrini D (2012). Long pentraxin 3/tumor necrosis factor-stimulated gene-6 interaction: a biological rheostat for fibroblast growth factor 2-mediated angiogenesis. Arterioscler Thromb Vasc Biol.

[65] Rodriguez-Grande B, Varghese L, Molina-Holgado F, Rajkovic O, Garlanda C, Denes A (2015). Pentraxin 3 mediates neurogenesis and angiogenesis after cerebral ischaemia. J Neuroinflammation.

[66] Woo JM, Kwon MY, Shin DY, Kang YH, Hwang N, Chung SW (2013). Human retinal pigment epithelial cells express the long pentraxin PTX3. Mol Vis.

[67] Yang HS, Woo JE, Lee SJ, Park SH, Woo JM (2014). Elevated plasma pentraxin 3 levels are associated with development and progression of diabetic retinopathy in Korean patients with type 2 diabetes mellitus. Invest Ophthalmol Vis Sci.

[68] Zhou W, Hu W (2016). Serum and vitreous pentraxin 3 concentrations in patients with diabetic retinopathy. Genet Test Mol Biomarkers.

[69] Min JK, Kim J, Woo JM (2015). Elevated plasma pentraxin3 levels and its association with neovascular age-related macular degeneration. Ocul Immunol Inflamm.

[70] Park KS, Kim JW, An JH, Woo JM (2014). Elevated plasma pentraxin 3 and its association with retinal vein occlusion. Korean J Ophthalmol.

[71] Alberti L, Gilardini L, Zulian A, Micheletto G, Peri G, Doni A (2009). Expression of long pentraxin PTX3 in human adipose tissue and its relation with cardiovascular risk factors. Atherosclerosis.

[72] Ogihara Y, Ogata S, Nomoto K, Ebato T, Sato K, Kokubo K (2014). Transcriptional regulation by infliximab therapy in Kawasaki disease patients with immunoglobulin resistance. Pediatr Res.

[73] Chick HE, Nowrouzi A, Fronza R, McDonald RA, Kane NM, Alba R (2012). Integrase-deficient lentiviral vectors mediate efficient gene transfer to human vascular smooth muscle cells with minimal genotoxic risk. Hum Gene Ther.

[74] Acevedo L, Yu J, Erdjument-Bromage H, Miao RQ, Kim JE, Fulton D (2004). A new role for Nogo as a regulator of vascular remodeling. Nat Med.

[75] Satomura K, Torigoshi T, Koga T, Maeda Y, Izumi Y, Jiuchi Y (2013). Serum amyloid A (SAA) induces pentraxin 3 (PTX3) production in rheumatoid synoviocytes. Mod Rheumatol.

[76] Preda MB, Ronningen T, Burlacu A, Simionescu M, Moskaug JO, Valen G (2014). Remote transplantation of mesenchymal stem cells protects the heart against ischemia-reperfusion injury. Stem Cells.

[77] Rodriguez-Grande B, Swana M, Nguyen L, Englezou P, Maysami S, Allan SM (2014). The acute-phase protein PTX3 is an essential mediator of glial scar formation and resolution of brain edema after ischemic injury. J Cereb Blood Flow Metab.

[78] Merchant S, Korbelik M (2013). Upregulation of genes for C-reactive protein and related pentraxin/complement proteins in photodynamic therapy-treated human tumor cells: enrolment of PI3K/Akt and AP-1. Immunobiology.

[79] Norata GD, Marchesi P, Pirillo A, Uboldi P, Chiesa G, Maina V (2008). Long pentraxin 3, a key component of innate immunity, is modulated by high-density lipoproteins in endothelial cells. Arterioscler Thromb Vasc Biol.

[80] Moalli F, Doni A, Deban L, Zelante T, Zagarella S, Bottazzi B (2010). Role of complement and Fc{gamma} receptors in the protective activity of the long pentraxin PTX3 against Aspergillus fumigatus. Blood.

[81] Juel HB, Faber C, Munthe-Fog L, Bastrup-Birk S, Reese-Petersen AL, Falk MK (2015). Systemic and ocular long pentraxin 3 in patients with age-related macular degeneration. PLoS One.

